# A novel mechanism of 6-methoxydihydroavicine in suppressing ovarian carcinoma by disrupting mitochondrial homeostasis and triggering ROS/ MAPK mediated apoptosis

**DOI:** 10.3389/fphar.2023.1093650

**Published:** 2023-05-05

**Authors:** Huachang Zhang, Fugen Shangguan, Lan Zhang, Nengfang Ma, Shuling Song, Li Ma, Chuntong Liu, Mengke Liu, Jing An, Hua Li, Qizhi Cao

**Affiliations:** ^1^ Department of Immunology, School of Basic Medical Sciences, Binzhou Medical University, Yantai, Shandong, China; ^2^ Key Laboratory of Diagnosis and Treatment of Severe Hepato-Pancreatic Diseases of Zhejiang Province, The First Affiliated Hospital of Wenzhou Medical University, Wenzhou, China; ^3^ The Affiliated Taian City Central Hospital of Qingdao University, Taian, Shandong, China; ^4^ School of Life and Environmental Sciences, Wenzhou University, Wenzhou, China; ^5^ School of Gerontology, Binzhou Medical University, Yantai, Shandong, China; ^6^ Division of Infectious Diseases and Global Health, School of Medicine, University of California San Diego (UCSD), La Jolla, CA, United States

**Keywords:** 6-methoxydihydroavicine (6-ME), ovarian cancer (OC), mitochondria homeostasis, reactive oxygen species (ROS), MAPK, oxaloacetic acid (OAA) metabolism

## Abstract

**Introduction:** Alkaloids derived from *M. cordata* (Papaveraceae family), have been found to display antineoplastic activity in several types of cancer. However, the antitumor effects and mechanisms of a new alkaloid extracted from the fruits of *M. cordata*, named 6-Methoxydihydroavicine (6-ME), remains unclear in the case of ovarian cancer (OC).

**Methods:** CCK-8 assay was employed to analyze the cell viabilities of OC cells. RTCA, and colony-formation assays were performed to measure OC cell growth. Alterations in apoptosis and ROS levels were detected by flow cytometry in accordance with the instructions of corresponding assay kits. A Seahorse XFe96 was executed conducted to confirm the effects of 6-ME on cellular bioenergetics. Western blot and q-RT-PCR were conducted to detect alterations in target proteins. The subcutaneous xenografted tumor model of OC was used to further validate the anti-tumor activity of 6-ME *in vivo*.

**Results:** Here, we reported for the first time that 6-ME inhibits OC cells growth *in vitro* and *in vivo*. Meanwhile, we found that 6-ME showed great antineoplastic activities by disrupting mitochondria homeostasis and promoting apoptosis in OC cells. Further investigation of the upstream signaling of apoptosis revealed that 6-ME-triggered apoptosis was induced by reactive oxygen species (ROS)-mediated mitogen-activated protein kinase (MAPK) activation and mitochondria dysfunction in OC cells. Furthermore, we found oxaloacetic acid (OAA), a crucial metabolite has been proved to be related to NADPH production, can block the cytotoxicity and accumulation of ROS caused by 6-ME in OC cells.

**Discussion:** In summary, our data show that 6-ME exhibits cytotoxicity to OC cells in a ROS-dependent manner by interrupting mitochondrial respiration homeostasis and inducing MAPK-mediated apoptosis. This evidence suggests that 6-ME is a promising remedy for OC intervention.

## 1 Introduction

Ovarian cancer (OC), a common gynecological tumor with a high mortality rate, is the eighth leading cause of morbidity and mortality among women worldwide, with 313,959 cases and 207,252 deaths in 2020 (Sung et al., 2021). Nearly 90% of OC cases are epithelial ovarian cancer (EOC) histotypes, main including high-grade serous, low-grade serous, endometrioid, clear cell, and mucinous subtypes (Torre et al., 2018; Millstein et al., 2020). High-grade serous ovarian cancer (HGSOC) accounts for more than 65% of EOC cases and contributes to the high mortality rate of EOC (Torre et al., 2018). Due to the difficulties of early diagnosis of EOC, about 70% of patients are already advanced at the time of diagnosis, with a 5-year survival rate of less than 50% (Torre et al., 2018; Herrera et al., 2019; Schoutrop et al., 2022). Currently, chemotherapy is still the main strategy for OC treatment, but the side effects and resistance of chemotherapy drugs remain urgent issues to be solved (Lisio et al., 2019; Marchetti et al., 2021). Therefore, the exploitation of novel and safety antitumor remedies of natural origin is of great importance. Fortunately, a range of natural compounds, likes alkaloids, terpenes, and polyphenols, have been shown to have therapeutic potential for cancer (Atanasov et al., 2021). *Macleaya cordata (Willd.) R. Br.* (*M. cordata*), a traditional medicine belongs to the *Papaveraceae* family that has long been used in Asia, especially in China to treat myodynia, wound inflammation, and bee stings (Ali et al., 2021). Previous studies have indicated that *M. cordata* contains a variety of alkaloids, which are the basis for demonstrating its medical benefits (Guo et al., 2014). The explorations of the potential pharmacological functions of these alkaloids seem to be valuable, particularly in the context of tumor treatment. To date, several *M. cordata* alkaloids have been found to exhibit antitumor activity in different types of cancer. For example, the *M. cordata* alkaloids sanguinarine, chelerythrine, and berberine have shown promising antineoplastic properties in pancreatic cancer (Almeida et al., 2017; Zhu et al., 2019; Ali et al., 2021). Chelerythrine triggered apoptosis in gastric cancer by reducing the expression of Bcl-xL and Bcl-2 proteins (Zhengfu et al., 2012). Macleayins A exerted its anti-cervical cancer activity by inhibiting Wnt/β-Catenin-dependent proliferation and inducing apoptosis (Sai et al., 2021). Ethoxysanguinarinenon regulated the AMPK/mTOR pathway as an antitumor property (Si et al., 2019). Given this evidence, further investigation of safe and effective natural compounds from these alkaloids for cancer intervention is practical and worth pursuing.

Many chemotherapeutic drugs exhibit antitumor properties by initialing programmed cell death (PCD), which includes necroptosis (Su et al., 2016; Jing et al., 2018), pyroptosis (Wang et al., 2017; Wu et al., 2021), ferroptosis (Liang et al., 2019; Lei et al., 2022), and apoptosis (Tang et al., 2019; Shahar and Larisch, 2020). As the most well-understood and extensively researched type of PCD, apoptosis occurs with membrane blebbing, nuclear fragmentation, and cell shrinkage (Koren and Fuchs, 2021). Reactive oxygen species (ROS), which are always initialed by oxidative stimuli and some antitumor drugs, have been shown to be closely associated with apoptosis (Jacquemin et al., 2015; Holze et al., 2018; Li et al., 2020; Jiang et al., 2022). Noticeably, MAPK signaling, main including JNK/MAPK, ERK/MAPK, and p38/MAPK proteins, has been found to act as a bridge for ROS-induced apoptosis. Mao et al. showed that shikonin triggers ROS/JNK-dependent apoptosis in leukemia cells (Mao et al., 2008). Curcumin derivatives have consistently been found to active apoptosis in breast cancer via the ROS/YAP/JNK axis (Wang et al., 2019). Moreover, Lan et al. observed that deferoxamine induces esophageal cancer cell apoptosis through ROS/ERK-reliant mitochondrial dysfunction (Lan et al., 2018). Furthermore, the ERK/p38-MAPK axis has been linked to ROS-initialed apoptosis caused by liposomal honokiol in medulloblastoma cells (Li C et al., 2022). Conversely, the activation of ERK/MAPK may have anti-apoptosis effects in some circumstances (Dang et al., 2017; Lu et al., 2021; Xing et al., 2021). Recent studies have indicated that δ-Tocotrienol increases the sensitivity of OC cells to cisplatin by facilitating the ROS/JNK and ROS/p38 pathways (Fontana et al., 2021). Similarly, Zhu et al. found that escin exhibited promising anti-osteosarcoma property by activating ROS/p38-dependent apoptosis (Zhu et al., 2017). All these discoveries suggest the important and unique role of the MAPK axis in ROS-induce apoptosis.

In this study, we report a novel alkaloid, 6-ME, with excellent anti-OC activity, which has never been published before. We focus on elucidating the underlying mechanisms by which 6-ME exhibits its antitumor properties in OC cells.

## 2 Materials and methods

### 2.1 Cell lines and cell culture

The CAOV3 and SKOV3 were brought from the Cell Bank of the Chinese Academy of Sciences (Shanghai, China). CAOV3 and SKOV3 cell lines were cultured in DMEM medium and McCoy’s 5A respectively, supplementing with 10% fetal bovine serum and penicillin-streptomycin. All cells were incubated in a humidified incubator with 5% CO_2_ at 37°C.

### 2.2 Reagents and antibodies

Reagents are listed in Supplementary Table S1 while antibodies are shown in Supplementary Table S2.

### 2.3 Cell viability assessment

The inhibitory concentration (IC_50_) of 6-ME was estimated by conducting CCK-8 assay in CAOV3 and SKOV3 cells according to the manufacturer’s instructions. The cells were counted and seeded at 10,000 cells per well in 96-well cell culture plates and cultured overnight. They were then t treated with concentration gradients of 6-ME or DMSO for 24 h, followed by an additionally 3 h incubation with CCK-8 solution at 37°C. Afterward, the observance at OD _450nm_ was measured using Varioskan Flash (Thermo Scientific).

### 2.4 Measurement of cell proliferation by real time cellular analysis (RTCA)

RTCA assays were conducted to measure the anti-proliferation activity of 6-ME in OC cells following the manufacturer’s protocol. The SKOV3 cells were counted and seeded at the density of 10,000 cells per well and cultured with the indicated concentrations of 6-ME at 37°C for several days. The data were then exported and graphed.

### 2.5 Colony formation assay

CAOV3 and SKOV3 cells were inoculated in 6-well cell culture plates at the density of 1,000/well and cultured under the presence or absence of different concentrations of 6-ME for 2 weeks. After washing three times with PBS, fixing for 30 min with 4% paraformaldehyde, and staining for 30 min with crystal violet, clonal colonies >40–50 cells/each were counted.

### 2.6 Western blot analysis

CAOV3 and SKOV3 cells were incubated with 6-ME alone or in combination with NAC or OAA for 24 h. These cells were then collected, incubated with appropriate cell lysis buffer on ice for 20 min, and centrifuged at 12, 000 rpm for 20 min at 4°C. After transferring the supernatants to a new 1.5 ml EP tube on ice, the protein concentration was determined using Pierce™ BCA Protein Assay kit (Thermo Fisher Scientific, 23,225) following the manufacturer’s protocol. Subsequently, the protein concentration was adjusted to 1 μg/μL/sample with 5X DualColor Protein Loading Buffer (Fude Biological Technology, FD006) and heated at 95°C for 5 min in a Digital Dry Baths/Block Heaters (Thermo Fisher Scientific, 88870005). For western blot analysis, protein samples (20 μg/each) were loaded onto the SDS-PAGE gel, electrophoresed, and transferred onto 0.22 μM PVDF membranes. Afterblocking with 5% NON-Fat Powdered Milk (Solarbio Life Science, D8340) for 90 min, the membranes were incubated with the desired primary antibodies overnight at 4°C, washed with 1X TBS-T for 5 min at least three time, and incubated with corresponding secondary antibodies at room temperature for 90 min. Finally, these membranes were washed with 1X TBS-T for 10 min at least three times, visualized with the SuperSignal™ West Pico PLUS kit (Thermo Fisher Scientific, 34,580), and quantified using ImageJ software.

### 2.7 Cell apoptosis analysis

CAOV3 and SKOV3 cells were incubated with different concentrations of 6-ME for 12 h, then harvested, and stained with Annexin V-FITC/PI at room temperature for 20 min in darkness, and detected by flow cytometry.

### 2.8 Photograph of cell morphology

After culturing CAOV3 and SKOV3 cells with 6-ME for 24 h, cell morphologies were viewed under a LEICA DMI1 microscope and photographed using a LAS V4.12 digital camera (LEICA Corporation) with a ×10 eyepieces and ×20 objective.

### 2.9 Oxygen consumption rate (OCR) measurement

The XFe96 extracellular flux analyzer was employed to measure the alterations of OCR when OC cells were exposed to 6-ME alone or in combination with NAC or OAA. The OC cells (20,000 cells per well) were seed in specialized cell plates and cultured overnight at 37°C. Afterward, the cells were exposed to 6-ME alone or 6-ME combined with NAC or OAA for 4 h; they were then incubated for another 1 h with a base medium containing Glucose and Pyruvic acid sodium. Finally, three working solutions, including Oligomycin, FCCP, and Rotenone/Antimycin A, were added to the corresponding wells, and changes in OCR were detected using the XFe96 Extracellular Flux analyzer under specific procedures.

### 2.10 Extracellular acidification rate (ECAR) measurement

The XFe96 extracellular flux analyzer was employed to measure the alterations of ECAR when OC cells were exposed to 6-ME. The OC cells (20,000 cells per well) were seed in specialized cell plates and cultured overnight at 37°C. Afterward, the cells were exposed to 6-ME for 4 h; they were then incubated for another 1 h with a base medium containing Glutamine. Finally, three working solutions, including Glucose, Oligomycin, and 2-DG were added to the corresponding wells, and changes in ECAR were detected using the XFe96 Extracellular Flux analyzer under specific procedures.

### 2.11 FACS analysis for ROS

To examine the alteration of reactive oxygen species (ROS) in 6-ME -treated OC cells for 12 h, we stained the cells with dichloro-dihydro-fluorescein diacetate (DCFH-DA) and analyzed them using flow cytometry in accordance with manufacturer’s protocol.

### 2.12 Measurement of MMP

To detect changes in mitochondrial membrane potential (MMP) in OC cells after 12 h treatment with 6-ME, we dyed the cells with JC-1 and analyzed them by flow cytometry according to the manufacturer’s protocol.

### 2.13 Nude mouse tumor-bearing experiments

All animal studies were carried out in accordance with the protocol approved by the Animal Protection and Utilization Committee of Binzhou Medical University. Female BALB/c-nude mice were raised in an environment free of specific pathogens (SPF). In 5-week-old nude mice (n = 8), CAOV3 cells (5×10^6^) were injected subcutaneously ventrally. The mice were separated into two groups of four mice each when the tumor volume reached ∼100 mm^3^. 5% DMSO, 40% PEG300, 5% Tween80, 50% H_2_O was used as the solvent of 6-ME. Mice in one group received an intraperitoneal injection of 6-ME (5 mg/kg), whereas mice in the control group received an injection of solvent. The tumor volume (mm^3^) was calculated using the formula volume = length × (width) ^2^/2 after the tumor size was determined using a vernier caliper. Every 2 days, weigh it. After 14 days, the mice were sacrificed and the tumors were photographed, dissected, and weighed.

### 2.14 Statistical analysis

All the statistical analyses were performed using SPSS16.0 statistical analysis software and GraphPad Prism 5. The data are shown as the mean ± SD of three independent replicate experiments. T-test was used to analyze the statistical difference between two independent groups and one-way ANOVA with the Tukey *post-hoc* test was used to analyze differences more than two groups for a single variable. *p*-value <0.05 was considered statistically significant.

## 3 Results

### 3.1 6-ME inhibits *in vitro* OC cell growth

**FIGURE 1:**
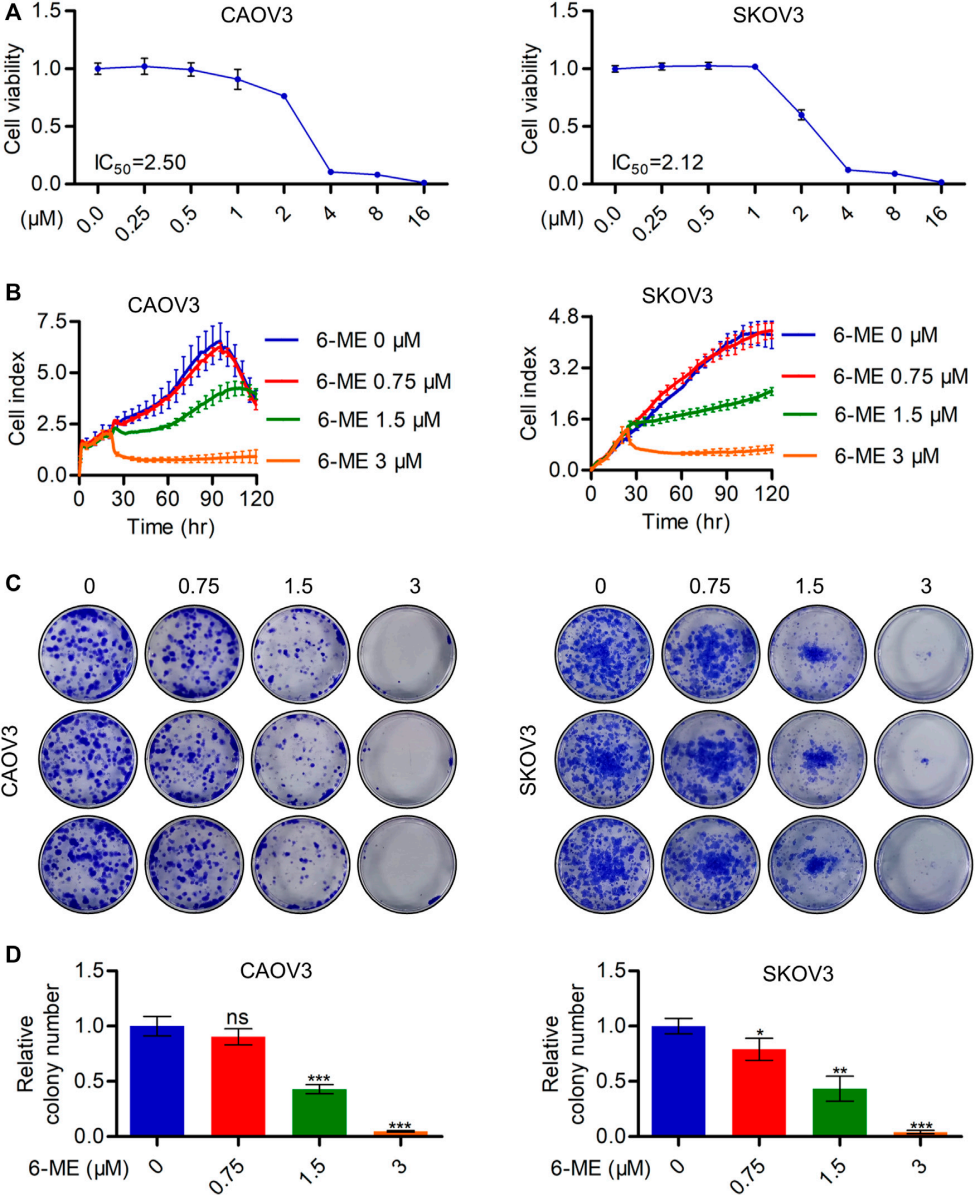
6-ME depresses *in vitro* OC cell growth. **(A)**. CCK-8 assays were used to analyze the inhibitory activities of 6-ME in OC cells. **(B)**. RTCA assays were performed to detect the ability of proliferation in OC cells. **(C)**. The clonogenic activities were confirmed by performing colony formation assays. **(D)**. The numbers of clonal colonies were counted and then analyzed, data was showed as mean ± SD.

The inhibitory activities of 6-ME on CAOV3 and SKOV3 cell survivals were initially detected using CCK-8 assays (Figure 1A). Subsequent RTCA assays revealed a significant decrease in the proliferation of OC cells after 6-ME exposure (Figure 1B). Reduction in the clonogenic activities in 6-ME-treated OC cells were further confirmed by performing colony formation assays (Figures 1C, D). These data indicate that 6-ME effectively represses the *in vitro* growth of OC cells.

### 3.2 6-ME triggers caspase-dependent apoptosis in OC cells

**FIGURE 2:**
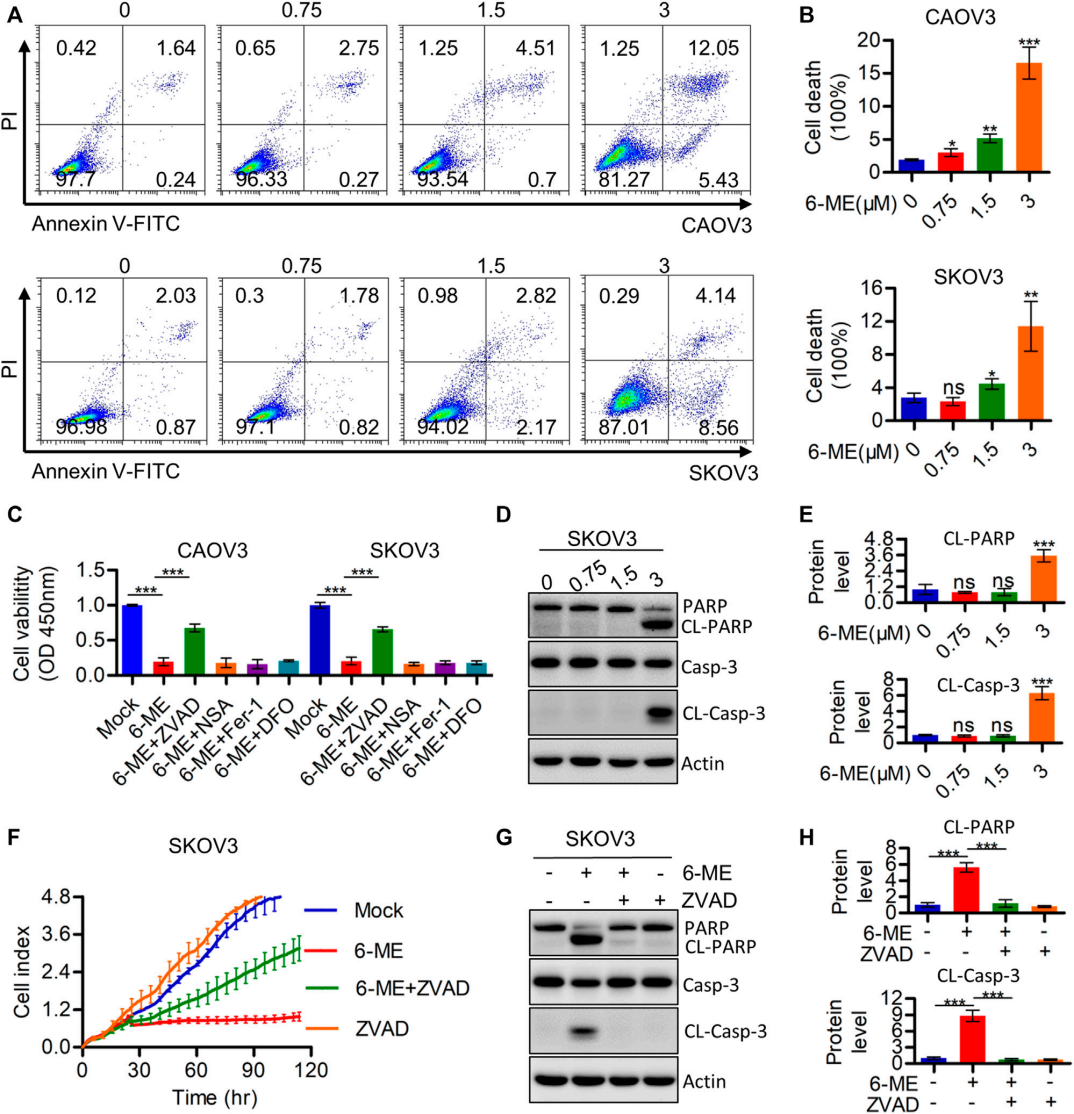
6-ME induces Caspase-mediated apoptosis in OC cells. **(A)**. The cell death was analyzed by flow cytometry in Annexin V-FITC/PI dyeing OC cells. **(B)**. The cell death rates were statistically analyzed and showed as mean ± SD. **(C)**. Cell viabities were measured after 6-ME treated alone or in combination with PCD inhibitors, data was showed as mean ± SD. **(D)**. Western blot were used to detect the activation of PARP and caspase-3, Actin was acted as a loading control. **(E)**. The cleavage of PARP and caspase-3 were quantified and statistically analyzed, data was showed as mean ± SD. **(F)**. RTCA assays were performed to detect the ability of proliferation in 6-ME exposed OC cells in the presence of ZVAD. **(G)**. Western blot were used to detect the activation of PARP and caspase-3 in 6-ME exposed OC cells in the presence of ZVAD, Actin was acted as a loading control. **(H)**. The cleavage of PARP and caspase-3 were quantified and statistically analyzed, data was showed as mean ± SD.

The cytotoxicity caused by 6-ME in OC cells was further investigated using flow cytometry. Annexin V-FITC/PI data showed that 6-ME significantly induced OC cell death when compared to DMSO vehicle control (Figures 2A, B). Subsequent measurements of cell viability after treatment with 6-ME alone or in combination with corresponding PCD inhibitors showed that the presence of the apoptosis inhibitor ZVAD, but not of other cell-death inhibitors, including necroptosis inhibitor NSA and ferroptosis inhibitors DFO or Fer-1, eliminated the cytotoxicity caused by 6–ME (Figure 2C). However, in 6-ME-exposed OC cells, we did not observe the appearance of balloon-like bubbles and cleavage of gasdermin family proteins (GSDMB, GSDMC, GSDMD, and GSDME), which are typical cell morphology of pyroptosis (Supplementary Figures S1A, B). Echoing the results of counteracting 6-ME-induced inhibition of cell proliferation (Figure 2F; Supplementary Figure S1C), treatment of OC cells with 6-ME in the presence or absence of Z-VAD halted the increase in 6-ME-mediated cleavages of PARP and caspase-3 (Figures 2D, E, G, H). These results show that 6-ME exerts its anti-OC properties by initializing caspase-dependent apoptosis.

### 3.3 6-ME impairs mitochondrial respiration and induces oxidative stress, but does not alter aerobic glycolysis in OC cells

**FIGURE 3:**
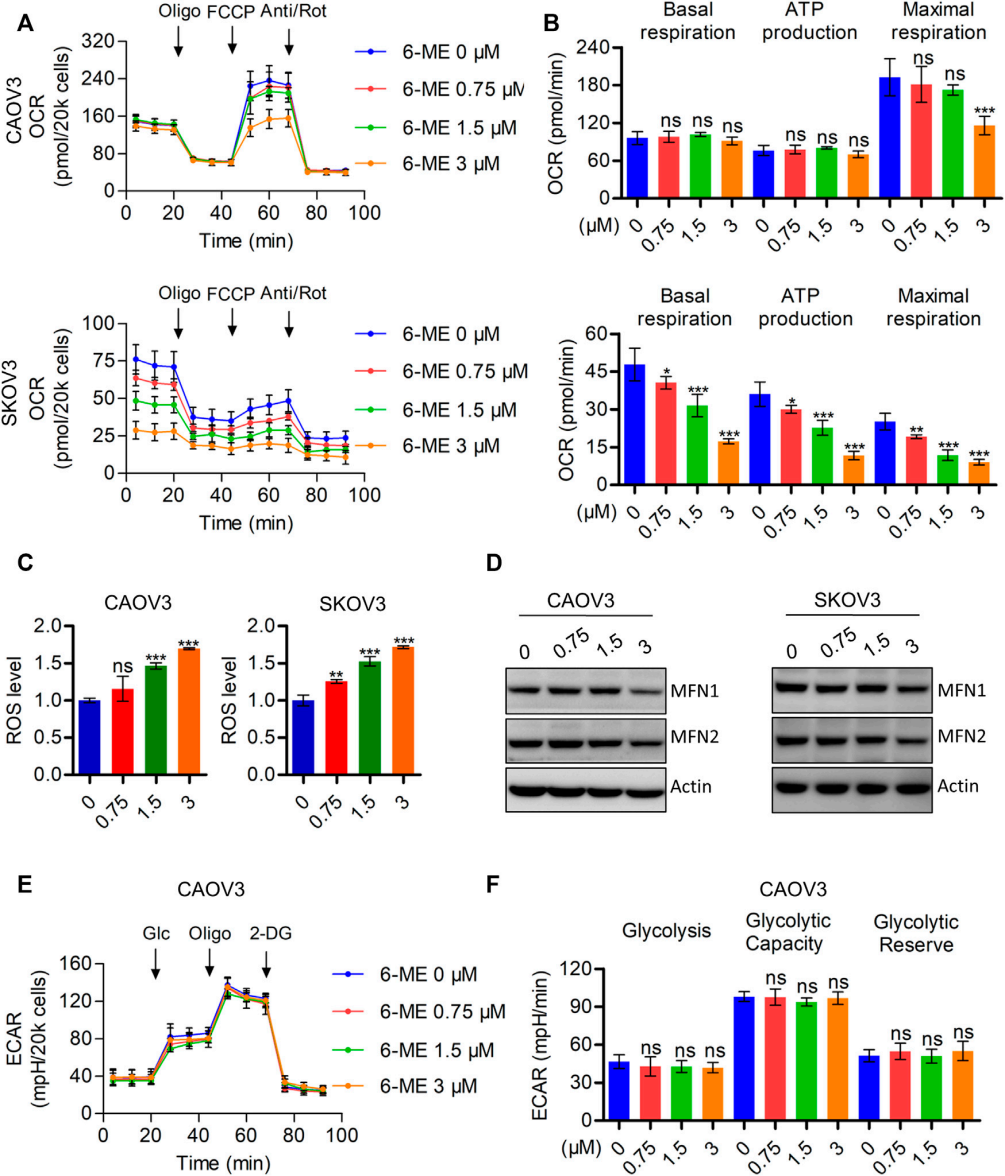
6-ME triggers ROS accumulation and mitochondrial dysfunction. **(A)**. The OCR was measured by Seahorse XF96 bioenergy analyzer after 6-ME addition. **(B)**. The data comes from OCR was analyzed and showed as basal respiration, ATP production, and maximal respiration, all data were showed as mean ± SD. **(C)**. ROS level was analyzed by flow cytometry in DCFH-DA staining OC cells and showed as mean ± SD. **(D)**. The alteration of MFN1 and MFN2 were confirmed by western blot, actin was acted as a loading control. **(E)**. The ECAR was measured by Seahorse XF96 bioenergy analyzer after 6-ME administration. **(F)**. The data comes from ECAR was analyzed and showed as glycolysis, glycolytic capacity, and glycolytic reserve, all data were showed as mean ± SD.

It is well known that disruption of mitochondrial function can cause caspase-3-dependent apoptosis. Therefore, we hypothesized that the interruption of mitochondria homeostasis by 6-ME is a step in its response that causes cytotoxicity in OC cells. To corroborate this hypothesis, we analyzed the oxygen consumption rate (OCR) after 6-ME administration using the Seahorse XF96 bioenergy analyzer. 6-ME significantly inhibited not only the total OCR but also the maximal respiration of CAOV3 and SKOV3 cells (Figures 3A, B). Moreover, 6-ME exposure resulted in increased in ROS levels (Figure 3C) and inhibition of the key mitochondrial fusion proteins MFN1 and MFN2 (Figure 3D; Supplementary Figure S2A). To further explore the effects of 6-ME on aerobic glycolysis, a crucial resource of ATP and metabolic intermediates for tumor growth, we analyzed the extracellular acidification rate (ECAR) using the Seahorse XF96 bioenergy analyzer. Our results reveal that 6-ME is unable to alter the hemostasis of aerobic glycolysis in OC cells (Figures 3E, F). These findings suggest that 6-ME initializes mitochondrial dysfunction and oxidative stress in OC cells.

### 3.4 6-ME exhibits its anti-OC properties by triggering mitochondrial dysfunction through the promotion of ROS production, which can be eliminated by the antioxidant NAC

**FIGURE 4:**
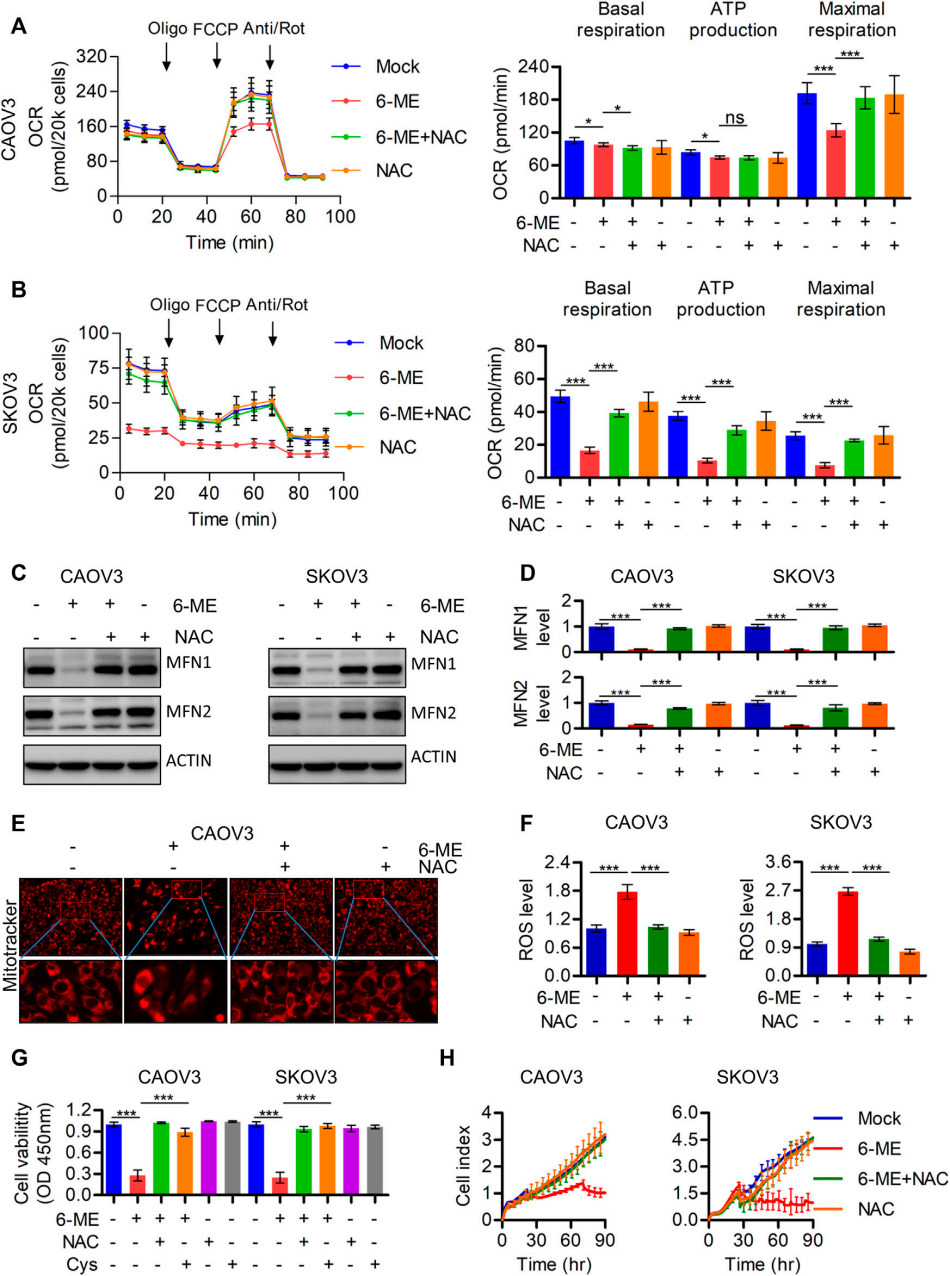
6-ME caused ROS activation leads to the dysfunction of mitochondria in OC cells. **(A,B)**. The OCR was measured by Seahorse XF96 bioenergy analyzer in 6-ME treated cell with or without NAC. **(C)**. The alteration of MFN1 and MFN2 were confirmed in 6-ME exposed OC cells in the presence of NAC by western blot, actin was acted as a loading control. **(D)**. The changes of MFN1 and MFN2 were quantified and statistically analyzed, data was showed as mean ± SD. **(E)**. 6-ME-caused mitochondrial morphological abnormalities were observed in Mitotracker dyed OC cells. **(F)**. ROS level was analyzed by flow cytometry in 6-ME treated OC cells after NAC addition and the data was showed as mean ± SD. **(G)**. The cell viabilities were analyzed in 6-ME exposed alone or combination of NAC or Cys and data was showed as mean ± SD. **(H)**. The cell proliferation abilities were analyzed in 6-ME exposed alone or combination of NAC and data was showed as mean ± SD.

To further investigate the underlying mechanism by which 6-ME regulates mitochondrial imbalance, the effects of ROS on mitochondrial homeostasis were subsequently determined. Exploration of 6-ME treated alone or combination with ROS scavenger NAC uncovered that NAC markedly eliminated the decrease in overall OCR and maximal respiration (Figures 4A, B). Moreover, the 6-ME-induced inhibitions of MFN1 and MFN2 expressions were alleviated by the addition of NAC (Figures 4C, D). 6-ME-caused mitochondrial morphological abnormalities were also markedly attenuated by NAC, based on the results of Mitotracker staining (Figure 4E). Culturing OC cells with NAC clearly eliminated the effects of 6-ME on promoting ROS accumulation in the cells (Figure 4F). NAC also mitigated the inhibitory effects of 6-ME on OC cell viability and proliferation (Figures 4G, H). Overall, 6-ME-induced mitochondrial dysfunction is mediated by ROS accumulation, which leads to OC cell growth inhibition, while the antioxidant NAC significantly overcomes the 6-ME-induced mitochondrial impairment.

### 3.5 6-ME causes ROS/MAPK axis-dependent apoptosis in OC cells

**FIGURE 5:**
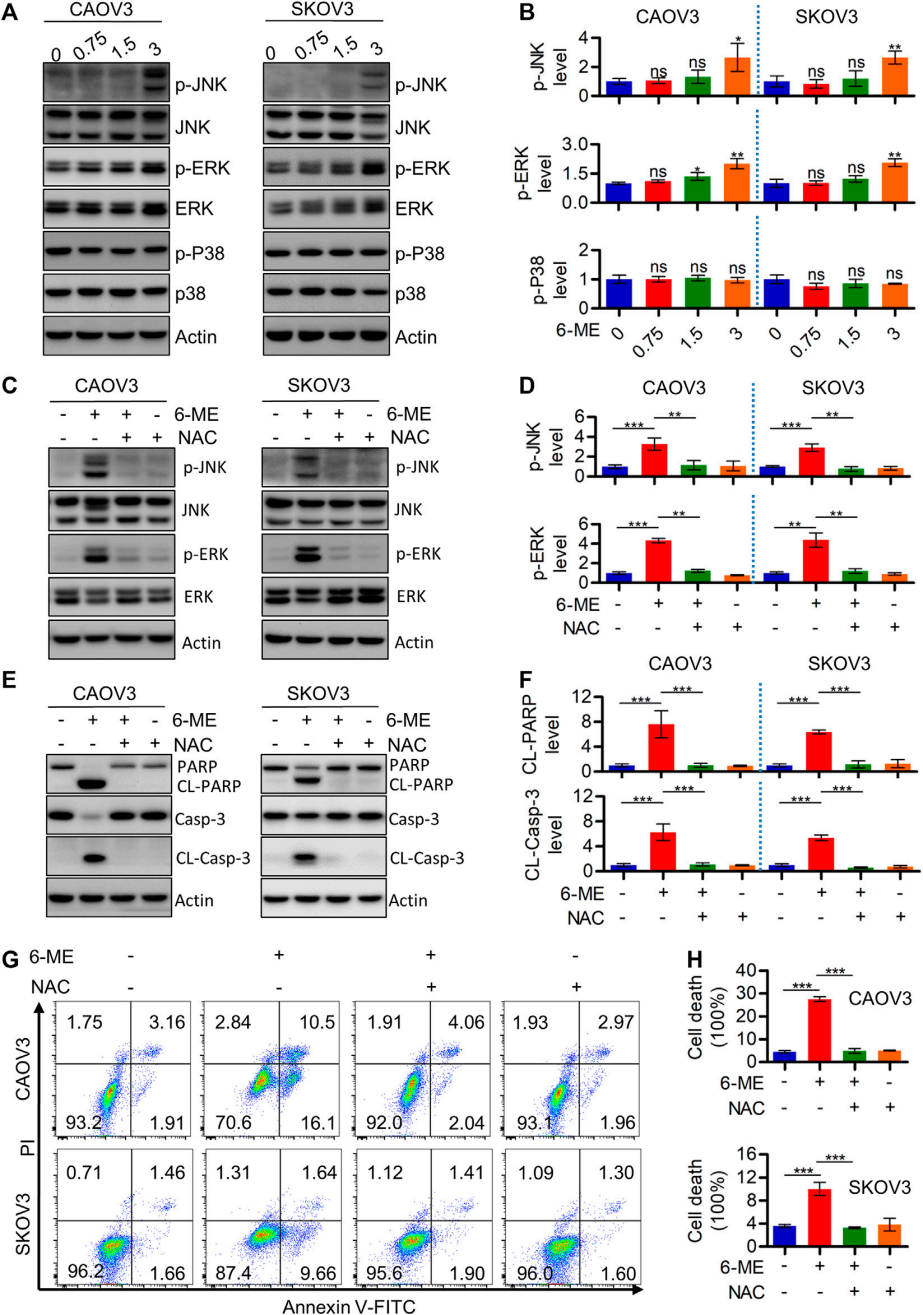
6-ME activates ROS/MAPK axis-dependent apoptosis in OC cells. **(A)**. Western blot was performed to detect the activation of JNK, ERK, and p38, the Actin was act as a loading control. **(B)**. The changes of these proteins were then quantified and analyzed, the data was showed as mean ± SD. **(C)**. Western blot was performed to detect the activation of JNK and ERK in 6-ME treated with or without NAC in OC cells, the Actin was act as a loading control. **(D)**. The changes of p-JNK and p-ERK were then quantified and analyzed, the data was showed as mean ± SD. **(E)**. Western blot was performed to detected the activation of PARP and caspase-3 in 6-ME treated with or without NAC in OC cells, the Actin was act as a loading control. **(F)**. The changes of PARP and caspase-3 were then quantified and analyzed, and the data was showed as mean ± SD. **(G)**. The cell death was analyzed by flow cytometry in Annexin V-FITC/PI dyeing and 6-ME cultured OC cells in the presence of NAC. **(H)**. The cell death rates were then statistically analyzed and showed as mean ± SD.

Considering the important role of ROS in modulating MAPK signaling, Western blotting was further performed to detect alterations of crucial factors involved in the pathway in response to 6-ME treatment. JNK/MAPK and ERK/MAPK, but not p-p38, were activated in OC cells after 6-ME treatment (Figures 5A, B). Culturing OC cells with 6-ME alone or in combination with NAC confirmed that the addition of NAC obviously diminished the effects of 6-ME on MAPK axis activation (Figures 5C, D). Similarly, 6-ME-promoted cleavages of PARP and caspase-3 were mitigated by NAC (Figures 5E, F). Measurement of the apoptotic rates in OC cells cultured with 6-ME alone or in combination with NAC further revealed that the presence of NAC led to the reduction of 6-ME-induced cell death (Figures 5G, H). These observations imply that 6-ME exerts its cytotoxicity by initiating apoptosis, at least in part, through ROS-mediated MAPK activation.

### 3.6 6-ME may disrupt the metabolic homeostasis of OAA and thereby activating ROS production

**FIGURE 6:**
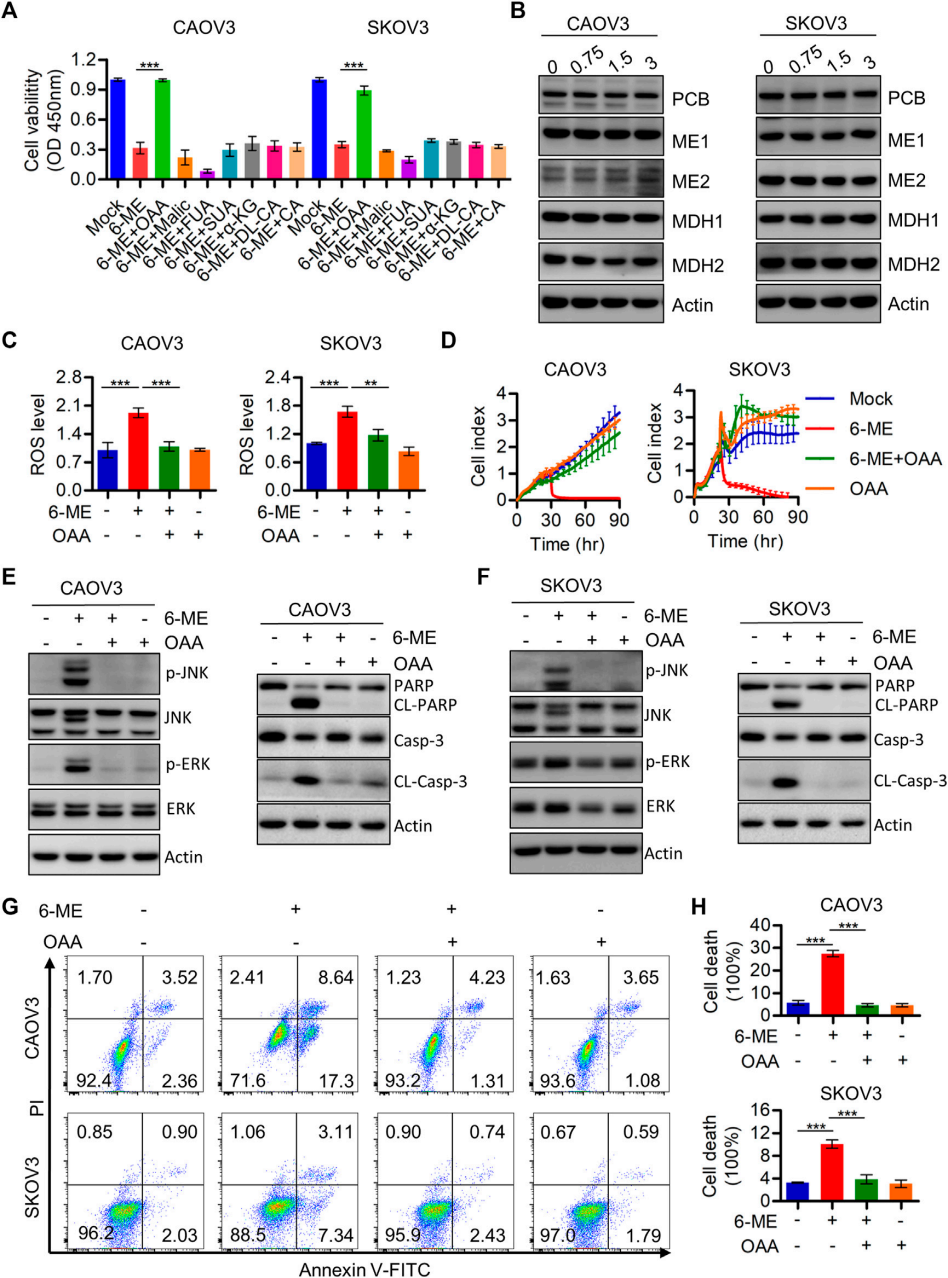
6-ME may disrupt the metabolic homeostasis of OAA and thereby activating ROS production. **(A)**. Cell viabilities were measured after 6-ME treated alone or in combination with TCA cycle metabolites, data was showed as mean ± SD. **(B)**. Western blot were used to detect the alteration of PCB, MDH1, MDH2, ME1 and ME2, Actin was acted as a loading control. **(C)**. ROS level was analyzed by flow cytometry in 6-ME treated OC cells after OAA addition and the data was showed as mean ± SD. **(D)**. The cell proliferation abilities were analyzed in 6-ME exposed alone or combination of OAA and data was showed as mean ± SD. **(E,F)**. Western blot was performed to detect the activation of JNK, ERK, PARP, and caspase-3 in 6-ME treated with or without OAA in OC cells, the Actin was act as a loading control. **(G)**. The cell death was analyzed by flow cytometry in Annexin V-FITC/PI dyeing and 6-ME cultured OC cells in the presence of OAA. **(H)**. The cell death rates were then statistically analyzed and showed as mean ± SD.

This study demonstrated the indispensable roles of ROS in regulating cell death and mitochondrial dysfunction. Therefore, the regulatory mechanisms behind the 6-ME-induced increase in ROS production were further explored. It is worth mentioning that oxaloacetic acid (OAA), but not other crucial metabolites of the TCA cycle counteracted the cytotoxicity caused by 6-ME in OC cells (Figure 6A). Considering the important role of OAA in regulating NADPH production, we also speculated that 6-ME could modulate ROS generation by disrupting OAA metabolic homeostasis. The alterations of crucial enzymes involved in OAA metabolism were thus analyzed by Western blotting, which confirmed that the expression of PCB (pyruvate carboxylase), MDH1, MDH2, ME1, ME2, the key enzymes for OAA synthesis, unchanged after 6-ME treatment (Figure 6B). Interestingly, detection of ROS levels in 6-ME-exposed cells with or without OAA revealed that OAA mitigated the activation of ROS after 6-ME addition (Figure 6C). The addition of OAA also effectively reverted 6-ME-induced proliferation inhibition (Figure 6D), eliminated the 6-ME-mediated increase in phosphorylation of JNK and ERK and cleavages of PARP and caspase-3 (Figures 6E, F; Supplementary Figures S4A, B), and mitigated the influence of 6-ME-promoted apoptosis in OC cells (Figures 6G, H). These results indicate that 6-ME may contribute to increased ROS levels by disturbing OAA metabolism homeostasis, leading to ROS/MAPK-dependent apoptosis in OC cells.

### 3.7 6-ME depress *in vivo* OC cell growth in nude mouse

**FIGURE 7:**
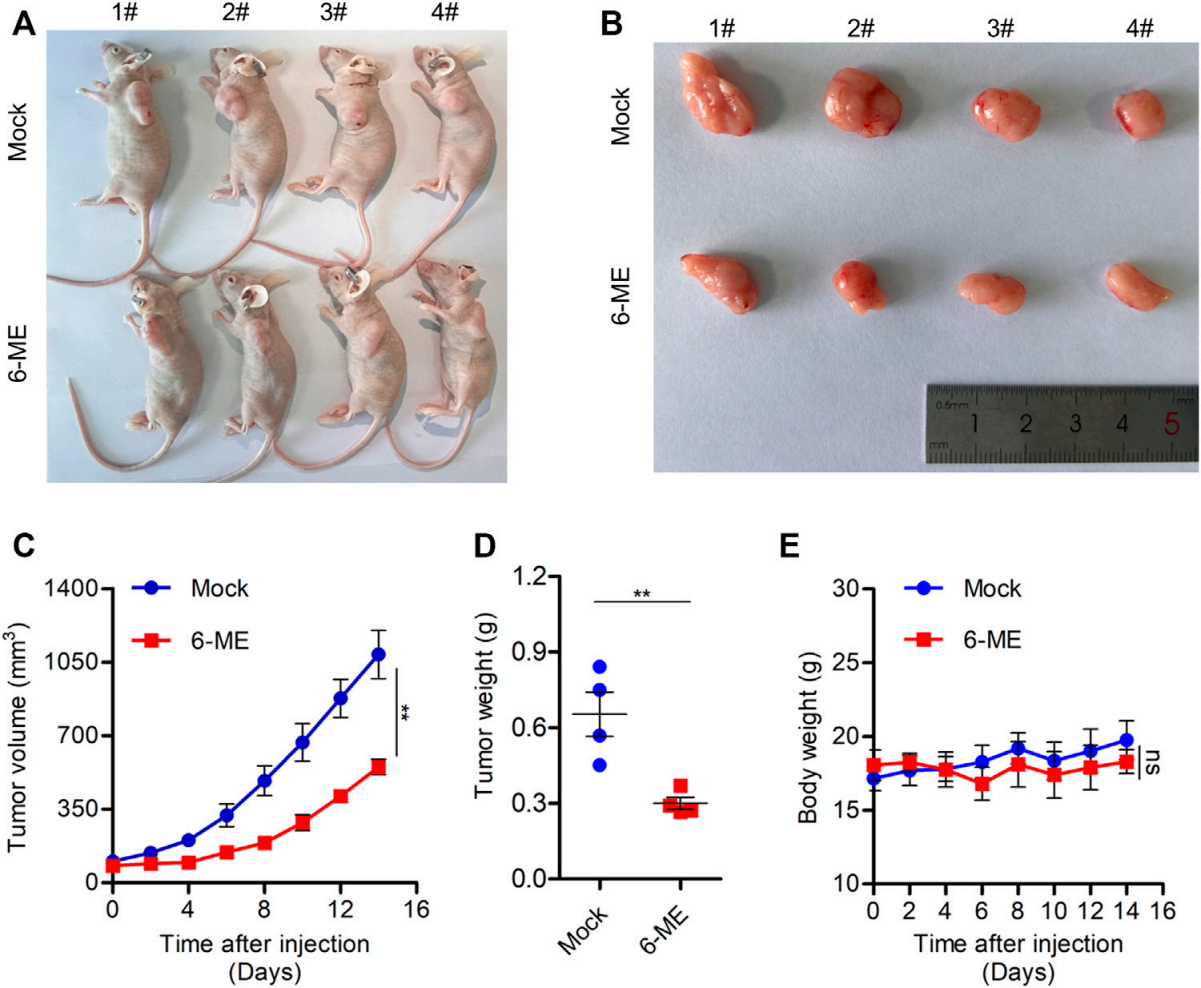
6-ME efficiently blocks OC growth *in vivo*. **(A)** Images of nude mice treated with 6-ME (5 mg/kg). **(B)** Corresponding tumor tissue pictures obtained after the nude mice were sacrificed. **(C)** Tumor growth in nude mice after treatment with 6-ME. **(D)** The tumor weight of OC treated with 6-ME was analyzed. **(E)** Volume changes of nude mice in two groups. The data showed as mean ± SD.

We used a tumor-bearing nude mouse model to further investigate the anti-tumor effect of 6-ME *in vivo*. 6-ME dramatically reduced tumor growth in tumor-bearing nude mice (Figures 7A, B), which is consistent with the experimental evidence of OC cells *in vitro*. In comparison to the control group, the 6-ME group’s tumor volume and weight were much lower (Figures 7C, D). Since there was no discernible change in body weight between the two groups, 6-ME showed no physiologically harmful effects on mice (Figure 7E). These findings imply that 6-ME is a potentially effective anticancer medication for the treatment of ovarian carcinoma.

## 4 Discussion

The promising antitumor properties of alkaloids and others extracts from *M. cordata* have been demonstrated in different cancer types (Zhengfu et al., 2012; Guo et al., 2014; Almeida et al., 2017; Si et al., 2019; Zhu et al., 2019; Ali et al., 2021; Sai et al., 2021). Notably, our recent study has confirmed that 6-ME can induce mitochondrial dysfunction and ROS/RIPK1-dependent pyroptosis that hinder pancreatic cancer progression (Ma et al., 2022). However, it remains unclear whether 6-ME possess a potential against OC and what the underlying mechanisms of its action might be. In the present study, we reported a novel anti-OC pathway in which 6-ME disrupts mitochondrial hemostasis and causes ROS/MAPK axis-dependent apoptosis. These findings indicate that 6-ME is a promising natural compound worthy of further investigation for OC intervention.

PCD, which primarily includes apoptosis, pyroptosis, necroptosis, and ferroptosis, has been employed in therapeutic strategies to kill cancer cells and/or hinder their progression (Koren and Fuchs, 2021). We employed corresponding PCD inhibitors to confirm the specific type of PCD in OC cells induced by 6-ME. The cell viability data showed that only the apoptosis inhibitor ZVAD, but not the other PCD inhibitors, can antagonize 6-ME-induced PCD (Figure 2C). Considering the cross talk between apoptosis and pyroptosis (Koren and Fuchs, 2021), we conducted further investigations to verify these processes. Western blotting results indicated that 6-ME could not to lead to increase in cleavages of GSDMB, GSDMC, GSDMD, and GSDME (Supplementary Figure S1B), which are crucial biochemical indexes of cellular pyroptosis. Also, no balloon-like bubbles were observed in 6-ME-exposed OC cells (Supplementary Figure S1A), which is the classical cell morphology of pyroptosis (Shi et al., 2017). Therefore, we confirm that 6-ME exerts its cytotoxicity by inducing OC cell apoptosis rather than GSDMX-mediated pyroptosis. Cleavages of PARP and caspase-3, two crucial factors of apoptosis, were initiated by 6-ME and inhibited upon the addition of ZVAD in OC cells (Figures 2G, H). The 6-ME-induced cytotoxicity was also mitigated by the presence of ZVAD, based on the viability data of OC cells treated with 6-ME alone or in combination with ZVAD (Figure 2F; Supplementary Figure S1C). Based on these results, we conclude that 6-ME triggers OC cell apoptosis, which is a different mode of cell death from the one previously reported in the 6-ME-induced pyroptosis of pancreatic cancer (Ma et al., 2022).

It is well known that avoidance and resistance to apoptosis are hallmarks of tumors, to fight cancer, we can use strategies that target them (Hanahan and Weinberg, 2011). In the past, many studies have demonstrated that targeting mitochondria-mediated apoptosis can be a candidate therapeutic option for cancer treatment (Burke, 2017). Our experimental results are consistent with this evidence by suggesting that 6-ME may exhibit cytotoxicity by modulating mitochondrial homeostasis and apoptosis in OC cells (Ma et al., 2019; Song et al., 2019; Temel et al., 2020). We found that 6-ME can dramatically inhibit mitochondrial respiration along with maximal respiration (Figures 3A, B). Furthermore, flow cytometry results revealed that 6-ME can remarkably trigger ROS production in OC cells (Figure 3C), providing that ROS is an important signal transduction that occurs in mitochondria-mediated apoptosis (Jacquemin et al., 2015; Li et al., 2019; Cao et al., 2020). Further investigations of the underlying mechanisms found that NAC, a potent ROS scavenger, can diminish the disruption of mitochondria and the activation of apoptosis caused by 6-ME when comparing OC cells exposed to 6-ME alone or in combination with NAC (Figures 4A, B; Figures 5G, H). This indicates that 6-ME may promote ROS-dependent mitochondrial dysfunction, thereby leading to the activation of OC cell apoptosis. Notably, ROS can further trigger the activation of some downstream pro-apoptotic signal pathways, such as the MAPK axis. MAPK has been reported as an important cellular signal transduction molecule that may act as a bridge between ROS and apoptosis in several cancer types (Mao et al., 2008; Dang et al., 2017; Zhu et al., 2017; Lan et al., 2018; Wang et al., 2019; Fontana et al., 2021; Lu et al., 2021; Xing et al., 2021; Li S et al., 2022). Similarly, in our study, we found that 6-ME-caused the activation of JNK/MAPK and ERK/MAPK in OC cells (Figures 5A, B). Moreover, the increase in phosphorylation of JNK/MAPK and ERK/MAPK, as well as increased cleavages of PARP and caspase 3 induced by 6-ME, can be eliminated by the addition of NAC (Figures 5C–F). Consistent with this, the 6-ME cause apoptosis was also blocked by NAC (Figures 5G, H). In particular, we observed an increase in 6-ME-induced phosphorylation of ERK/MAPK in the mitochondrial fraction (Supplementary Figure S3C), where they may behave as apoptotic inducers by causing mitochondrial dysfunction (Lan et al., 2018; Li C et al., 2022). These findings imply that 6-ME can induce the activation of JNK-ERK/MAPK to trigger subsequent apoptosis in OC cells.

Mitochondria are one of the main sources of cellular ROS production, and mitochondrial disruption can often occur during cancer development and treatment (Choucair et al., 2022; Dakik et al., 2022; Liu et al., 2022). We speculated that 6-ME-induced OC cell apoptosis is due to its involvement in triggering signals for ROS production and accumulation. To investigate this hypothesis, we cultured OC cells with 6-ME in the presence or absence of OAA (a crucial metabolite of the TCA cycle), which has been demonstrated to modulate the homeostasis of mitochondrial function (Desideri et al., 2015; Martinez-Reyes and Chandel, 2020). We thus confirmed that OAA, rather than other metabolites, can eliminate the cytotoxicity caused by 6-ME in OC cells (Figure 6A), which was consistent with our latest results regarding the effects of 6-ME and OAA on pancreatic cancer (Ma et al., 2022). The deficiency of OAA has been verified to contribute to the impairment of the balance of NADPH generation, which leads to the accumulation of ROS (Son et al., 2013; Abrego et al., 2017). Our subsequent studies further proved that the addition of OAA significantly impeded 6-ME-induced ROS accumulation (Figure 6C), JNK/MAPK and ERK/MAPK activation (Figures 6E, F), and increased apoptosis in OC cells (Figures 6G, H). Importantly, 6-ME has been reported that has the potential to bind to the regulatory center of enzymatic activity of PCB, which has been demonstrated to be the key enzyme supplying OAA to the TCA cycle (Ma et al., 2022). Meanwhile, we did not observe changes in PCB expression in 6-ME-treated OC cells by western blot (Figure 5B), suggesting that 6-ME may bind to PCB and then inhibit its activity rather than expression. The mechanism behind this process remains unclear and requires further investigation. Overall, 6-ME may modulate ROS production by directly binding to PCB and thereby hindering its activity.

**FIGURE 8:**
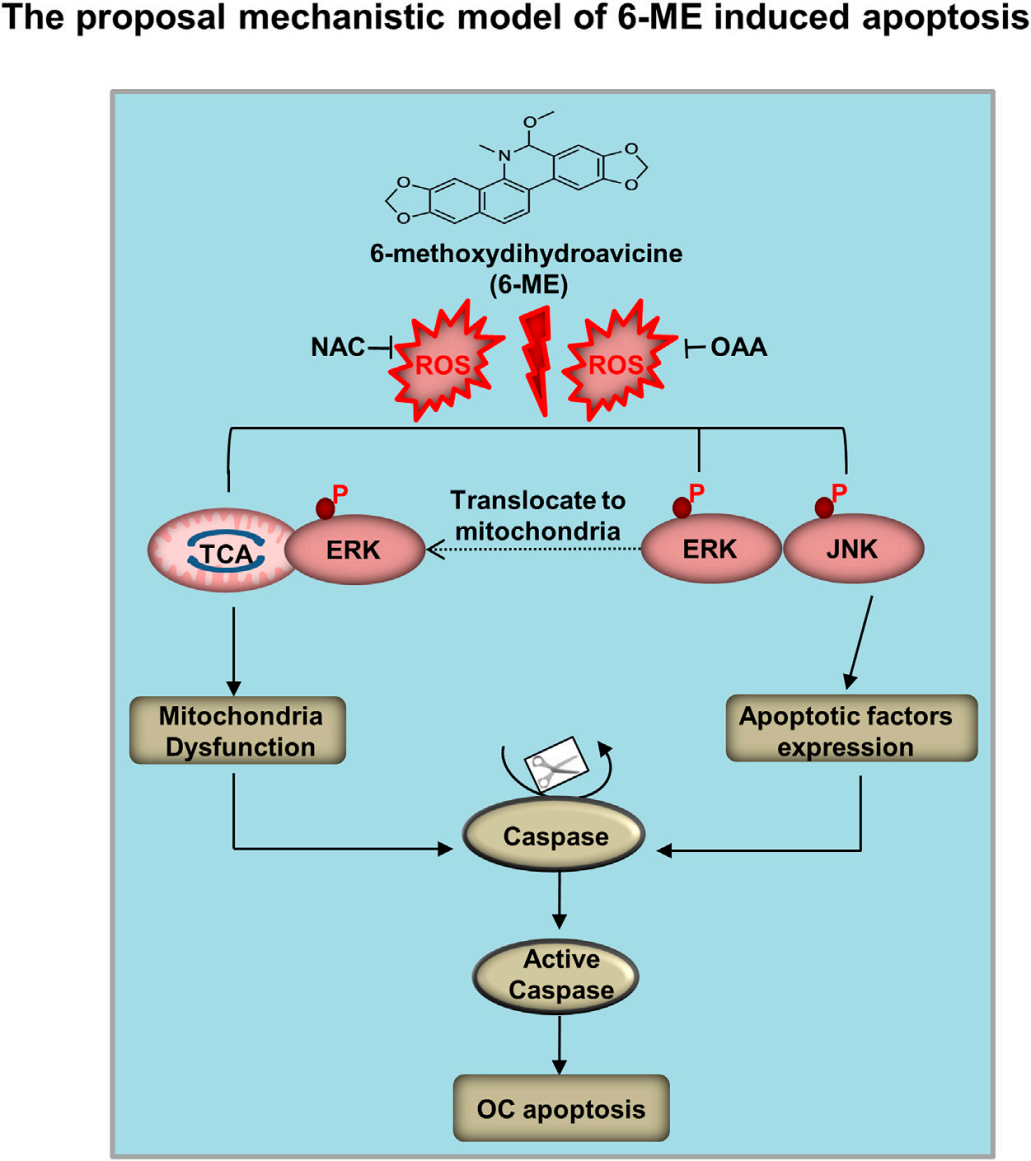
Mechanistic model of 6-ME induces OC cell apoptosis and mitochondrial dysfunction. 6-ME caused disruption of OAA metabolism and led to the accumulation of ROS. Subsequently, 6-ME caused ROS-facilitated JNK-ERK/MAPK activation and disruption of mitochondrial hemostasis, thus driving OC cell apoptosis.

In this study, we proposed a mechanistic model by which 6-ME inhibits cell growth and promotes apoptosis of OC cells *in vitro* and *in vivo* (Figure 8). 6-ME modulated PCB activity, which then caused disruption of OAA metabolism and led to the accumulation of ROS. Subsequently, 6-ME caused ROS-facilitated JNK-ERK/MAPK activation and disruption of mitochondrial hemostasis, thus driving OC cell apoptosis. These findings uncover novel mechanisms for the antineoplastic property of 6-ME in OC cells, supporting its potential as a natural drug for OC intervention. A 6-ME-based treatment approach may also offer significant benefits to patients with other tumors.

## Data Availability

The original contributions presented in the study are included in the article/Supplementary Material, further inquiries can be directed to the corresponding authors.
