# Supplementary material for: A novel mechanism of 6-methoxydihydroavicine in suppressing ovarian carcinoma by disrupting mitochondrial homeostasis and triggering ROS/ MAPK mediated apoptosis
Source: Front Pharmacol. 2023 May 5;14:1093650. doi: 10.3389/fphar.2023.1093650 (PMC10196025; doi:10.3389/fphar.2023.1093650)
Supplement: Supplementary file 1 [file Table1.DOCX]

Supplemental table 1

The list of reagents used in this study.

| Name | Cat No. | Company | Conutry |
| --- | --- | --- | --- |
| HRP-labeled Goat Anti-Rabbit IgG(H+L) | A0208 | Beyotime Biotechnology | China |
| HRP-labeled Goat Anti-Mouse IgG(H+L) | A0216 | Beyotime Biotechnology | China |
| Penicillin-Streptomycin Solution | C0222 | Beyotime Biotechnology | China |
| Z-VAD-FMK | C1202 | Beyotime Biotechnology | China |
| Cell Counting Kit-8 | C0037 | Beyotime Biotechnology | China |
| Reactive Oxygen Species Assay Kit | S0033 | Beyotime Biotechnology | China |
| Crystal Violet Staining Solution | C0121 | Beyotime Biotechnology | China |
| BSA, Fraction V | ST023 | Beyotime Biotechnology | China |
| Western Stripping buffer | P0025 | Beyotime Biotechnology | China |
| N-Acetyl-L-cysteine (NAC) | ST1546 | Beyotime Biotechnology | China |
| Cell Mitochondria Isolation Kit | C3601 | Beyotime Biotechnology | China |
| OAA | A600678 | BBI Life Science | China |
| Annexin V−FITC/PI apoptosis detection kit | AB_2869085 | BD Biosciences | USA |
| Trypsin | 03-050-1ACS | Biological Industries | USA |
| 5X DualColor Protein Loading Buffer | FD006 | Fude Biological Technology | China |
| DMEM medium | C11995500BT | Gbico | USA |
| RIPM-1640 medium | C11875500BT | Gbico | USA |
| Fetal bovine serum (FBS) | 10270-106 | Gbico | USA |
| Deferoxamine mesylate (DFO) | HY-B0988 | MedChemExpress | USA |
| Ferrostatin-1 (Fer-1) | HY-100579 | MedChemExpress | USA |
| Immobilon®-PSQ PVDF membrane | ISEQ00010 | Millipore | USA |
| PhosSTOP EASYpack | 04906837001 | Roche Applied Science | USA |
| Necrostatin-1 (NEC-1) | S8037 | Selleck Chemicals | USA |
| Necrosulfonamide (NSA) | S8251 | Selleck Chemicals | USA |
| 1 X PBS | P1020 | Solarbio Life Science | China |
| L-Cysteine (Cys) | C0012 | Solarbio Life Science | China |
| Puromycin | P8320 | Solarbio Life Science | China |
| 20X TBST | T1082 | Solarbio Life Science | China |
| NON-Fat Powdered Milk | D8340 | Solarbio Life Science | China |
| 30% Acr-Bis (29:1) | A1010 | Solarbio Life Science | China |
| Glycine | G8200 | Solarbio Life Science | China |
| SDS | S8010 | Solarbio Life Science | China |
| Tris (Hydroxymethyl) Aminomethane | T8060 | Solarbio Life Science | China |
| 1.5M Tris-HCL (pH 8.8) | T1010 | Solarbio Life Science | China |
| 1M Tris-HCl (pH 6.8) | T1020 | Solarbio Life Science | China |
| Glucose | G7021 | Sigma | USA |
| L-glutamine | GB0224 | [Sangon](https://www.sigmaaldrich.com/china-mainland.html) Biotech | China |
| Seahorse XF Cell Energy Phenotype Test Kit | 103325-100 | Seahorse Bioscience | USA |
| Seahorse XF Base Medium | 102353-100 | Seahorse Bioscience | USA |
| 6-Methoxydihydroavicine (6-ME) | S0906 | Selleck Chemicals | USA |
| Pierce^TM^ BCA protein assay kit | 23225 | Thermo Fisher Scientific | USA |
| SuperSignal™ West Pico PLUS Kit | 34580 | Thermo Fisher Scientific | USA |
