# Supplementary material for: A novel mechanism of 6-methoxydihydroavicine in suppressing ovarian carcinoma by disrupting mitochondrial homeostasis and triggering ROS/ MAPK mediated apoptosis
Source: Front Pharmacol. 2023 May 5;14:1093650. doi: 10.3389/fphar.2023.1093650 (PMC10196025; doi:10.3389/fphar.2023.1093650)
Supplement: Supplementary file 2 [file Table2.DOCX]

Supplemental table 2

A list of antibodies used in this study.

| Name | Cat No. | species | Company |
| --- | --- | --- | --- |
| GSDMD | ab210070 | Rabbit | Abcam |
| GSDME | ab215191 | Rabbit | Abcam |
| GSDMB | A7474 | Rabbit | Abcam |
| GSDMC | A14550 | Rabbit | ABclonal |
| β-Actin | ACO26 | Rabbit | ABclonal |
| MDH1 | A9673 | Rabbit | ABclonal |
| MDH2 | A6297 | Rabbit | ABclonal |
| ME1 | A3956 | Rabbit | ABclonal |
| ME2 | A9650 | Rabbit | ABclonal |
| CL-Caspase-3 | A11021 | Rabbit | ABclonal |
| PARP | #9542 | Rabbit | Cell Signaling Technology |
| Caspase-3 | #9662 | Rabbit | Cell Signaling Technology |
| p-JNK | #4668 | Rabbit | Cell Signaling Technology |
| p-ERK | #4370 | Rabbit | Cell Signaling Technology |
| p-P38 | #4511 | Rabbit | Cell Signaling Technology |
| ERK | #4695 | Rabbit | Cell Signaling Technology |
| JNK | 66210-1-AP | Mouse | Protein technology Group |
| P38 | 66234-1-AP | Mouse | Protein technology Group |
| MFN1 | 13798-1-AP | Rabbit | Protein technology Group |
| MFN2 | 12186-1-AP | Rabbit | Protein technology Group |
| TOM20 | 11802-1-AP | Rabbit | Protein technology Group |
| PCB | 16588-1-AP | Rabbit | Protein technology Group |
