# Supplementary material for: A novel mechanism of 6-methoxydihydroavicine in suppressing ovarian carcinoma by disrupting mitochondrial homeostasis and triggering ROS/ MAPK mediated apoptosis
Source: Front Pharmacol. 2023 May 5;14:1093650. doi: 10.3389/fphar.2023.1093650 (PMC10196025; doi:10.3389/fphar.2023.1093650)
Supplement: Supplementary file 3 [file DataSheet1.docx]

**A novel mechanism of 6-Methoxydihydroavicine in suppressing ovarian carcinoma by disrupting mitochondrial homeostasis and triggering ROS/ MAPK mediated apoptosis.**

Huachang Zhang^a,†^, Fugen Shangguan^b,†^, Lan Zhang ^c,†^, Nengfang Ma^d^, Shuling Song^a^, Li Ma^a^, Chuntong Liu^a^, Mengke Liu^a^, Jing An^e*^, Hua Li^c,^*, Qizhi Cao^a,^*

^a^Binzhou Medical University, Yantai 264003, Shandong, P.R. China.

^b^Key Laboratory of Diagnosis and Treatment of Severe Hepato-Pancreatic Diseases of Zhejiang Province, the First Affiliated Hospital of Wenzhou Medical University, Wenzhou 325000, China.

^c^Taian City Central Hospital, Taian 271000, Shandong,P.R. China.

^d^School of Life and Environmental Sciences, Wenzhou University, Wenzhou 325000, China.

^e^Division of Infectious Diseases and Global Health, School of Medicine, University of California San Diego (UCSD), LaJolla, CA92037, USA.

†These authors contributed equally to this work.

*Corresponding to Prof. QIzhi Cao, E-mail address: qizhicao@bzmc.edu.cn; Prof. Hua Li, E-mail address: yilulihua626@sina.com.

**Materials and method**

**Cell lines and cell culture**

The CAOV3 and SKOV3 were brought from the Cell Bank of the Chinese Academy of Sciences (Shanghai, China). CAOV3 and SKOV3 cell lines were cultured in DMEM medium and McCoy's 5A respectively, supplementing with 10% fetal bovine serum and penicillin-streptomycin. All cells were incubated in a humidified incubator with 5% CO_2_ at 37°C.

**Reagents and antibodies**

Reagents are listed in **Supplementary Table 1** while antibodies are shown in **Supplementary Table 2**.

**Photograph of cell morphology**

After culturing CAOC3 and SKOC3 cells with 6-ME for 24 h, cell morphologies were viewed under a LEICA DMI1 microscope and photographed using a LAS V4.12 digital camera (LEICA Corporation) with a 10x eyepieces and 20x objective.

**Western blot analysis**

CAOV3 and SKOV3 cells were incubated with 6-ME alone or in combination with NAC or OAA for 24 h. These cells were then collected, incubated with appropriate cell lysis buffer on ice for 20 min, and centrifuged at 12, 000 rpm for 20 min at 4°C. After transferring the supernatants to a new 1.5 mL EP tube on ice, the protein concentration was determined using Pierce^TM^ BCA Protein Assay kit (Thermo Fisher Scientific, 23225) following the manufacturer’s protocol. Subsequently, the protein concentration was adjusted to 1 μg/μL/sample with 5X DualColor

Protein Loading Buffer (Fude Biological Technology, FD006) and heated at 95°C for 5 min in a Digital Dry Baths/Block Heaters (Thermo Fisher Scientific, 88870005). For western blot analysis, protein samples (20 μg/each) were loaded onto the SDS-PAGE gel, electrophoresed, and transferred onto 0.22 μM PVDF membranes. Afterblocking with 5% NON-Fat Powdered Milk (Solarbio Life Science, D8340) for 90 min, the membranes were incubated with the desired primary antibodies overnight at 4°C, washed with 1X TBS-T for 5 min at least three time, and incubated with corresponding secondary antibodies at room temperature for 90 min. Finally, these membranes were washed with 1X TBS-T for 10 min at least three times, visualized with the SuperSignal™ West Pico PLUS kit (Thermo Fisher Scientific, 34580), and quantified using ImageJ software.

**Measurement of cell proliferation by real time cellular analysis (RTCA)**

RTCA assays were conducted to measure the anti-proliferation activity of 6-ME in OC cells following the manufacturer’s protocol. The CAOV3 cells were counted and seeded at the density of 10,000 cells per well and cultured with the indicated concentrations of 6-ME at 37°C for several days. The data were then exported and graphed.

**Isolation, extraction, and identification of mitochondria**

Mitochondria were isolated using the cell mitochondria isolation kit (C3606, Beyotime) according to the manufacturer’s instructions. Briefly, the mock-treated and 6-ME-treated CAOV3 cells well collected and suspended in the supplied isolation reagent and allowed to stand for 10 min at 4 °C. The mixture was then ground (approximately 100 times) and centrifuged at 750 g for 5 min at 4 °C. The supernatant was collected and re-centrifuged 9 000g for 15 min at 4 °C. The precipitated crude mitochondrial preparation was suspended in the supplied mitochondrial protective reagent and stored at -80 °C before western blotting analysis.

**Statistical analysis**

All the statistical analyses were performed using SPSS16.0 statistical analysis software and GraphPad Prism 5. The data are shown as the mean ± SD of three independent replicate experiments. T-test was used to analyze the statistical difference between two independent groups and one-way ANOCA with the Tukey *post-hoc* test was used to analyze differences more than two groups for a single variable. *P*-value < 0.05 was considered statistically significant.

**Results**

**Figure S1. A.** After culturing CAOC3 and SKOC3 cells with 6-ME for 24 h, cell morphologies were viewed under a LEICA DMI1 microscope and photographed using a LAS V4.12 digital camera (LEICA Corporation) with a 10x eyepieces and 20x objective. **B.** Western blot was performed to detect the activation of GSDMB, GSDMDC, GSDMD, and GSDME in 6-ME treated OC cells and pancreatic cancer cells (Positive control), the Actin was act as a loading control. **C.** RTCA assays were performed to detect the ability of proliferation in 6-ME exposed OC cells in the presence of ZVAD.

**Figure S2. A.** Western blot was performed to detect the activation of MFN1 and MFN2 in 6-ME treated OC cells, the data was quantified and showed as mean ± SD.

**Figure S3. A**. Western blot was performed to detect the expressions of JNK, ERK, and p38 in response to 6-ME treatment, the Actin was act as a loading control. The changes of these proteins were then quantified and analyzed, the data was showed as mean ± SD**. B.** Western blot was performed to detected the changes of JNK and ERK in 6-ME treated with or without NAC in OC cells, the Actin was act as a loading control. The changes of JNK and ERK were then quantified and analyzed, the data was showed as mean ± SD**. C.** Western blot was performed to detect the changes of p-ERK of mitochondrial fraction in 6-ME treated cells, TOM20 as a mitochondrial loading control and Actin as a cytoplasmic loading control respectively.

**Figure S4. A and B.** Western blot was performed to detected the activation of JNK, ERK, PARP, caspase-3 and the total protein pf JNK and ERK in 6-ME treated with or without OAA in OC cells, and the data was then quantified and showed as mean ± SD.
